# Amelioration of Reproduction-Associated Oxidative Stress in a Viviparous Insect Is Critical to Prevent Reproductive Senescence

**DOI:** 10.1371/journal.pone.0087554

**Published:** 2014-04-24

**Authors:** Veronika Michalkova, Joshua B. Benoit, Geoffrey M. Attardo, Jan Medlock, Serap Aksoy

**Affiliations:** 1 Department of Epidemiology of Microbial Diseases, Yale School of Public Health, New Haven, Connecticut, United State of America; 2 Section of Molecular and Applied Zoology, Institute of Zoology, Slovak Academy of Sciences, Bratislava, Slovakia; 3 Department of Biomedical Sciences, Oregon State University, Corvallis, Oregon, United States of America; Kansas State University, United States of America

## Abstract

Impact of reproductive processes upon female health has yielded conflicting results; particularly in relation to the role of reproduction-associated stress. We used the viviparous tsetse fly to determine if lactation, birth and involution lead to damage from oxidative stress (OS) that impairs subsequent reproductive cycles. Tsetse females carry an intrauterine larva to full term at each pregnancy cycle, and lactate to nourish them with milk secretions produced by the accessory gland ( = milk gland) organ. Unlike most K-strategists, tsetse females lack an apparent period of reproductive senescence allowing the production of 8–10 progeny over their entire life span. In a lactating female, over 47% of the maternal transcriptome is associated with the generation of milk proteins. The resulting single larval offspring weighs as much as the mother at birth. In studying this process we noted an increase in specific antioxidant enzyme (AOE) transcripts and enzymatic activity at critical times during lactation, birth and involution in the milk gland/fat body organ and the uterus. Suppression of *superoxide dismutase* (*sod*) decreased fecundity in subsequent reproductive cycles in young mothers and nearly abolished fecundity in geriatric females. Loss of fecundity was in part due to the inability of the mother to produce adequate milk to support larval growth. Longevity was also impaired after *sod* knockdown. Generation of OS in virgin females through exogenous treatment with hydrogen peroxide at times corresponding to pregnancy intervals reduced survival, which was exacerbated by *sod* knockdown. AOE expression may prevent oxidative damage associated with the generation of nutrients by the milk gland, parturition and milk gland breakdown. Our results indicate that prevention of OS is essential for females to meet the growing nutritional demands of juveniles during pregnancy and to repair the damage that occurs at birth. This process is particularly important for females to remain fecund during the latter portion of their lifetime.

## Introduction

Reactive oxygen species (ROS) are produced by mitochondrial respiration, which can be exacerbated during metabolic dysfunction, or as part of an immune response to pathogens. These factors can negatively interact with other biological molecules leading to oxidative damage [1]–[6]. According to the free-radical theory of aging, an organism that is unable to prevent or repair oxidative stress (OS), accumulates damage which leads to organismal dysfunction, aging and death [7]. This theory hypothesizes that presence of antioxidant enzymes (AOEs) is associated with increased longevity [6], [8]–[10]. However, recent studies have questioned the significance of the OS response on aging, and instead have suggested that other factors, such as dysregulation of nutrient signaling pathways, impaired proteolysis or reduced autophagy may be more important, with OS playing only a minor role in relation to aging [11]–[15]. In addition, the production of low levels of ROS appears critical to longevity and metabolic health by acting on intracellular signaling molecules at times of stress [16]. An alternative hypothesis that encompasses both of these ideas is that under optimal conditions ROS plays a role in stress signaling and may even have a positive effect in the prevention of aging. However, under sub-optimal conditions, or periods of high stress, individuals may not be able to mount an adequate response to high levels of ROS [13],[17],[18], leading to cellular damage and accelerated senescence.

Reproductive processes (pregnancy, parturition, lactation, and involution) have been documented to cause oxidative damage in mammalian systems [19]–[24]. The prevention of OS by AOE expression is critical to fecundity. In *Drosophila*, mosquitoes and sand flies knockdown of AOEs leads to reduced egg production [8], [9], [25]. Tsetse flies undergo viviparous reproduction, which deviates from the norm of oviparity that occurs in most insects. Tsetse females develop a single oocyte per gonotrophic cycle, and nurture a single offspring in their uterus during embryonic and larval development [26]. Unlike other K-strategists however, tsetse females don't exhibit an apparent period of reproductive senescence. Instead, tsetse females remain fertile throughout their adult lifetime to allow production of 8–10 progeny. During pregnancy, a single larva is nourished in the uterus by the milk secretions from the modified female accessory gland (referred to as milk or uterine gland) [26]. The milk gland undergoes involution (regression and breakdown of milk gland cells to pre-lactation levels) at the completion of each pregnancy cycle [27]. Nearly 20–25 mg of nutrients, consisting of a lipid-protein emulsion within an aqueous base, are transferred to each progeny during the 5–6 day period of intrauterine larval development [27], [28]. Over 50% of maternal lipid reserves are metabolized to provide the lipids and amino acids required for milk synthesis [29]–[32]. Larval progeny weigh as much as the mother at the time of birth. The fact that tsetse flies have such a heavy metabolic investment in their progeny makes the lack of reproductive senescence even more intriguing [33]. In addition, there is no observed difference in longevity between mated (reproductively active) and unmated tsetse females [33]. Other than a few specific examples, such as the naked mole rat [34], female fecundity typically declines or ceases with age. Furthermore, maternal investment in offspring development has other potential negative consequences on the mother, such as a reduction in longevity [35], [36]. Little is known about the processes tsetse females utilize to prevent and repair damage that occurs during lactation and intrauterine larval development to maintain the ability to produce progeny late in life.

In this study, we investigated the role of the lactation, birth and involution processes upon reproductive senescence and longevity using the viviparous tsetse fly system. We particularly focused on interplay between OS generation and the AOE response in relation to fecundity and longevity as well as on how females compensate for reproduction-induced stress to maintain high progeny output late in life. Our results support the role of the antioxidant response as a critical mechanism to prevent the premature reproductive senescence that results from the induction of OS during tsetse reproduction/lactation/birth processes. We discuss the implications of our findings in light of the interactions between reproduction, OS and antioxidant dynamics, and senescence in organisms with heavy nutritional investment in their progeny, including mammals and birds.

## Results

### Milk Gland Protein Synthesis is High During Lactation and Declines Immediately After Birth

**Figure 1:**
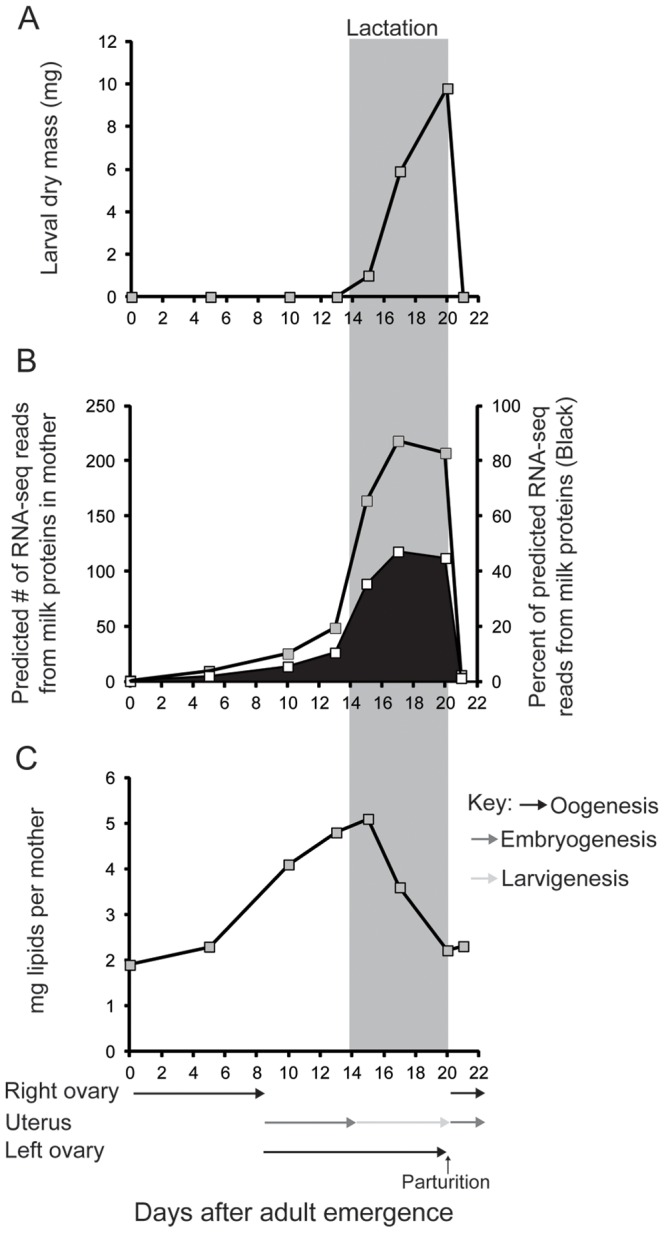
Tsetse fly investment in their progeny during lactation. A. Changes in dry mass of single intrauterine larva throughout development. B. Predicted read abundance for the 12 major milk protein genes (*milk gland protein 1–10*, *transferrin* and *acid sphingomyelinase 1*) throughout lactation based on fold changes in milk proteins in relation to transcriptome analysis measured at the peak of lactation (17–18 d) and 24–48 h after parturition according to Benoit et al. [40]. C. Total lipid content in females through pregnancy.

To provide a synopsis of the physiological changes that occur during the tsetse lactation and birth processes, we examined intrauterine larval size, maternal milk protein transcript abundance, and maternal lipid levels during pregnancy. Larval development occurs within the mother's uterus over a 5–6 day period. At the time of parturition the mature larva has a dry mass of over 10 mg (wet mass of 20–22 mg; Figure 1a), which is equivalent to the mass of the mother [27]. Predicted transcript levels for the twelve major milk proteins, which include Acid sphingomyelinase 1 (aSMase1 [37]), Transferrin (Trf [38]), a lipocalin (Milk Gland Protein 1; MGP1 [39]) and other milk proteins (MGP2-10 [40], [41]) account for over 47% of the total number of predicted RNA-seq reads in a library generated from lactating females carrying a mature intrauterine larva (Figure 1b). Within 24–48 h post-parturition, expression levels for all twelve major milk proteins dramatically decline to dry (non-lactating) levels where the major milk protein transcripts account for only 2% of the total reads (Figure 1b). In addition, the lipid reserves of females decline by over 50% during the later stages of the lactation period at each pregnancy cycle (based on Attardo et al. [29] and Figure 1c). The lipid breakdown that occurs at each pregnancy cycle is critical for the generation of diacylglycerol and proline [26], [42], [43], which are the major circulating nutrients in tsetse hemolymph and required for milk generation [30], [31], [44]. During pregnancy tsetse females appear to devote almost half of their nutritional/transcriptional investment towards production of milk for larval development. This process is under tight transcriptional regulation, with milk synthesis shut down within 24–48 hour post parturition [1].

### Lack of Increased OS Markers during Tsetse Lactation, Birth and Involution

**Figure 2:**
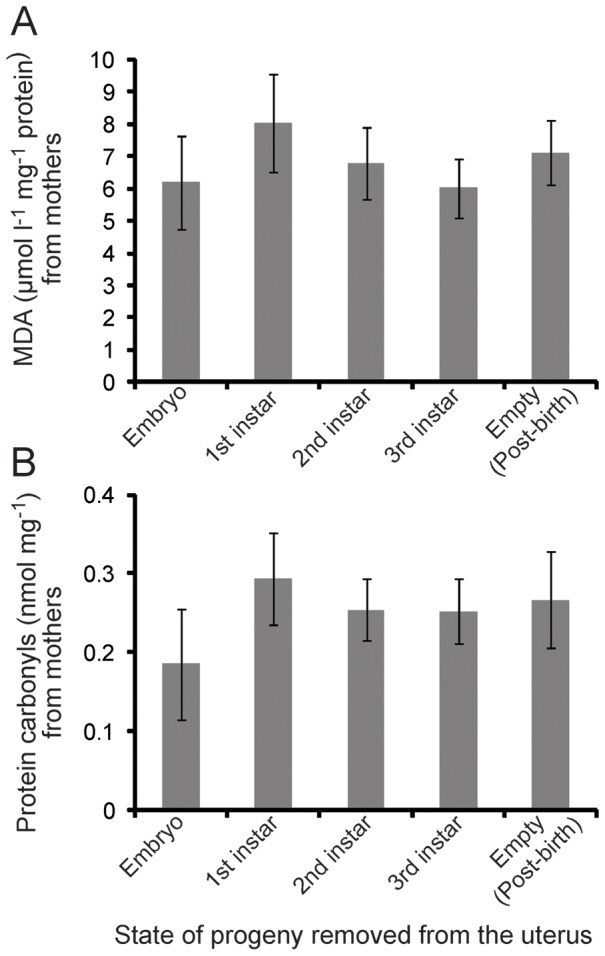
Levels of oxidative stress markers recovered from mothers through the 1^st^ gonotrophic cycle. A. Lipid oxidation levels by measurement of lipid peroxidation. Samples were collected from female flies after progeny removal throughout reproduction. Mean ± SE of five groups of 3 flies. B. Protein oxidation by measurement of protein carbonyl levels. Mean ± SE of five groups of 3 flies.

To determine if there is an increased level of oxidative damage throughout the tsetse pregnancy and birth cycle, we measured levels of two types of oxidative damage, protein carbonyls (proteins damaged by OS) and malondialdehyde (MDA; marker of lipid peroxidation), throughout reproduction. We found no difference in lipid peroxidation or protein carbonyls levels between pregnant mothers harboring an (embryo, 1^st^, 2^nd^ or 3^rd^ instar larva) and mothers immediately after birth (Figure 2). Our results indicate that there is no significant oxidative damage that results from the substantial physiological changes associated with viviparous reproduction in tsetse.

### Antioxidant (AOE) Gene Expression Increases during Lactation, Birth and Involution

**Figure 3:**
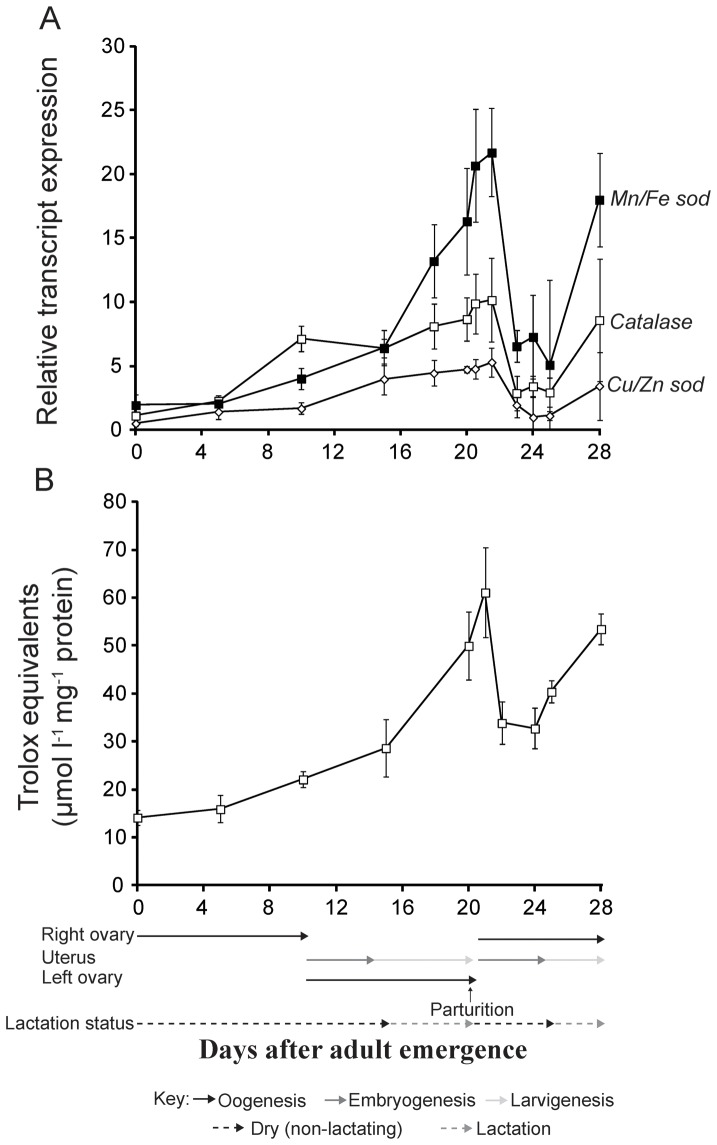
Antioxidant gene expression and activity levels throughout tsetse pregnancy. A. Transcript levels for *Mn/Fe superoxide dismutase* (*Mn/Fe sod*), *Cu/Zn sod* and *catalase* measured 24 h after the last blood meal. Each point represents the mean ± SE of four measurements. B. Antioxidant activity. Each sample represents the mean ± SE of four samples.

We next measured transcript levels for nine genes that code for antioxidant enzymes throughout the first and the beginning of the second reproductive cycle to determine if lack of OS could result from increased AOE expression. Transcript levels for 6 of the 9 genes did not vary throughout pregnancy significantly (Table S2). However, there was a substantial increase in the expression of *Cu/Zn sod*, *Mn/Fe sod* and *catalase* genes during lactation, and this expression profile continued up to 24–48 h post parturition during milk gland involution (Figure 3a). The expression of all three AOE genes declined to their lowest level 48–72 h after parturition before increasing again during the second pregnancy cycle (Figure 3a). The AOE activity followed a similar pattern to that of the AOE gene expression with higher antioxidant activity detected during the latter periods of lactation continuing until 24–48 hours post parturition (Figure 3b).

Due to the drastic changes in transcript abundance during pregnancy, *Cu/Zn sod*, *Mn/Fe sod* and *catalase* expression levels were measured before and during lactation, as well as during the involution period post parturition in different tissues of the mother, including fat body/milk gland and uterus (Figure S1a–e). These analyses allowed us to determine that the substantial increase in the AOE response during the tsetse reproductive cycle is localized to the fat body/milk gland and uterine tissues. AOE transcripts were found to increase in the fat body/milk gland and reproductive tract during lactation and involution (Figure S1a–c). A similar pattern was observed in regards to antioxidant activity with increased levels observed in the fat body/milk gland (Figure S1d). Separation of pure milk gland tissue is nearly impossible in tsetse females as it is intertwined with the fat body and trachea. Thus our dissections and measurements included both fat body and milk gland tissues. However, fluorescent *in situ* hybridization (FISH) analysis with gene specific primers indicates that the expression of both *Mn/Fe sod* and *Cu/Zn sod* occurs within the milk gland organ (Figure S2). These results show a substantial increase in AOE gene transcript levels and enzyme activity in response to lactation, birth and involution that is predominantly localized in tissues associated with larval growth and lactation.

### Reduction of SOD Expression Impairs Lactation and Results in Loss of Fecundity

To determine the functional role of the AOE response during tsetse reproduction, we utilized RNA interference to suppress the transcript levels of *sod* genes. Injection of siRNA targeting *Cu/Zn sod* and *Mn/Fe sod* yielded a reduction in transcript abundance of over 65% for both genes, respectively (Figure S3a). Antioxidant enzymatic activity was also significantly reduced by 20–30% when individual genes were silenced, and was further suppressed by combined interference of both SOD genes by 60–70% (Figure S3b). We next evaluated the ability of pregnant females injected with both si*Cu/Zn sod* and si*Mn/Fe sod* to survive an exogenous H_2_O_2_ treatment, as an indication of their ability to respond to OS. We found that knockdown of SOD genes rendered reproductively active females over 50% less tolerant to H_2_O_2_ treatment as measured by survival in relation to control counterparts (Figure S3c). It appears that the two most abundant AOE gene products (*Cu/Zn sod* and *Mn/Fe sod*) likely enable flies to cope with pregnancy associated OS.

**Figure 4:**
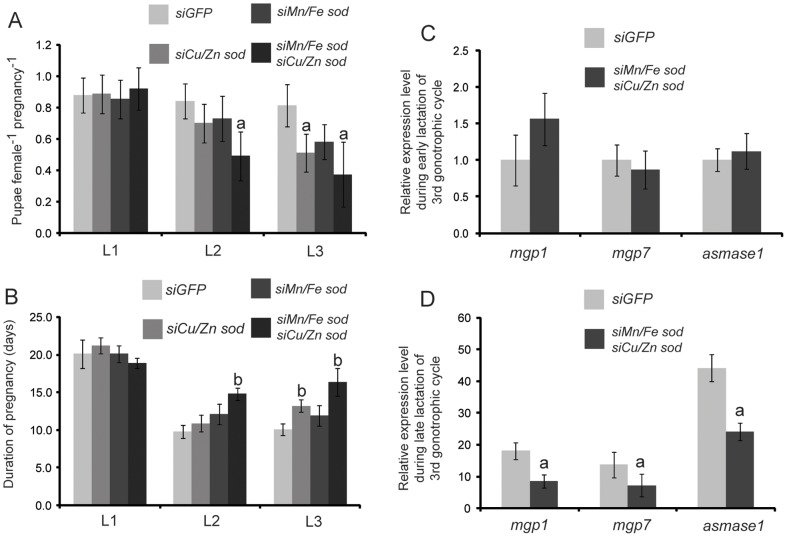
Effect of RNA interference of *Mn/Fe sod* and *Cu/Zn sod* on tsetse fecundity. A. Average number of pupae produced per female during generation of the 1^st^ (L1), 2^nd^ (L2) and 3^rd^ (L3) pregnancy cycle after *sod* knockdown in the 14^th^ day of the fly development and 5^th^ day of subsequent pregnancy cycles. Mean ± SE of three groups of 15 flies. B. Length of the gonotrophic cycles analyzed under similar treatment as A. Mean ± SE of three groups of 15 flies are shown. Expression of *milk gland proteins* (*mgp1*, *mgp7* and *asmase1*) during the early (C, 1^st^ instar larva present in uterus) and late stages (D, 3^rd^ instar larva present in uterus) of lactation after *sod* knockdown during the first two pregnancy cycles.

**Figure 5:**
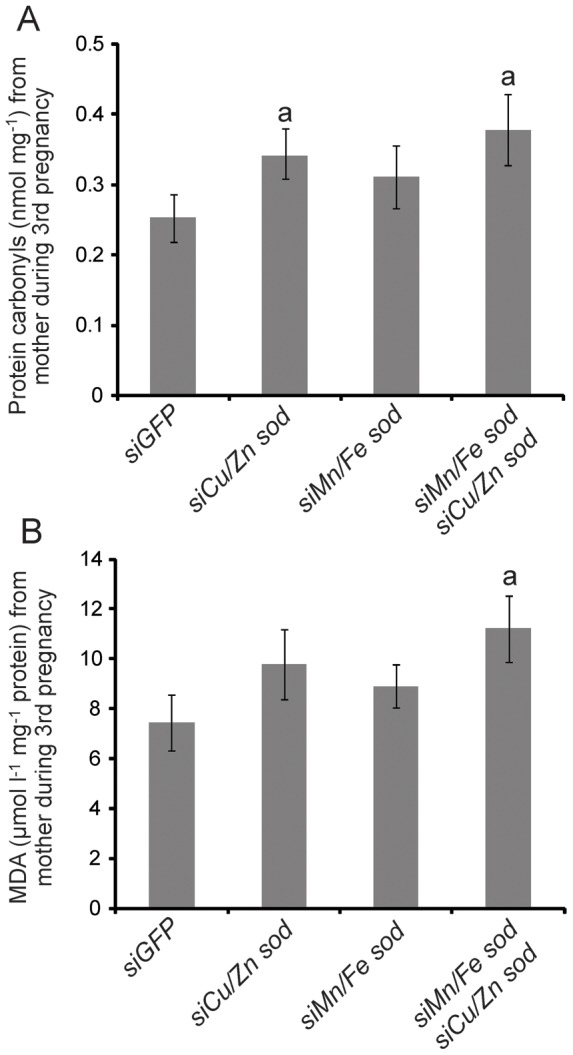
Oxidative stress markers recovered from females during their third pregnancy following *sod* knockdown during the first two reproductive cycles. A. Protein oxidation by measurement of protein carbonyl levels. Mean ± SE of five groups of 3 flies. B. Lipid oxidation levels measured by lipid peroxidation. Samples were collected from mothers carrying a 3^rd^ instar larvae in their uterus. Mean ± SE of five groups of 3 flies.

After demonstrating effective reduction of the antioxidant response by SOD gene knockdown, we assessed the effect of this phenotype on reproduction and lactation over the first three reproductive cycles in tsetse females by measuring progeny output. Knockdown of *Cu/Zn* or *Mn/Fe sod* individually during the first pregnancy cycle did not cause a significant reduction in pupal production during the next cycle relative to the control group that received siGFP treatments (Figure 4a). The combined knockdown of *Cu/Zn* and *Mn/Fe sod* in the first pregnancy cycle resulted in a slight reduction in progeny production in the second cycle (Figure 4a). However, the reduction of *Cu/Zn sod* alone during both the first and second cycles yielded nearly a 30% loss of fecundity during the third cycle (Figure 3a). Combined knockdown of both SOD genes during the first two cycles resulted in the lowest fecundity levels during the third cycle with a reduction of over 50% (Figure 4a). In addition to reduced progeny output, the length of the larval intrauterine development period was also extended by 15–20% following knockdown of a single SOD gene, and by over 30% when both SOD genes were suppressed in the earlier lactation cycles relative to the control treatment group (Figure 4b). The levels of protein carbonyl and markers of lipid peroxidation were also increased during subsequent pregnancy cycles following *sod* knockdown (Figure 5a and b). Collectively, our results indicate that SOD expression during periods of lactation and birth appears to be critical for the prevention of oxidative damage, and for the maintenance of tsetse's fecundity during subsequent reproductive cycles.

To determine the underlying aspects that lead to extended larval development period in SOD knockdown flies, we measured the transcript abundance of the three major milk proteins (MGP1, MGP7 and aSMase1) during the third pregnancy cycle after siRNA injection in the previous two pregnancy cycles (either *siGFP* or *siMn/Fe* and *siCu/Zn sod*). We noted no significant differences in the expression levels of the milk protein genes between the two groups when analyzed early in the lactation period when a first instar larva was present in the uterus (Figure 4c). However, there was a 2–3 fold reduction in milk protein gene expression levels later in lactation when a second or third instar larva was present in the uterus and milk production is normally peaking (Figure 4d). Along with reduced transcript levels of three major milk proteins, we documented a 30–35% decrease in protein level for the major milk protein MGP1 after SOD knockdown (Figure S4). These results indicate that the reduced fecundity observed in the older females after SOD knockdown is likely due to the reduced levels of milk proteins generated by the milk gland, which is insufficient to support adequate larval development.

### Reduction of Lactation-Associated AOEs Reduces Longevity and Development of Reproductive Senescence: Comparison with Reproductive Output in Other Animals

**Figure 6:**
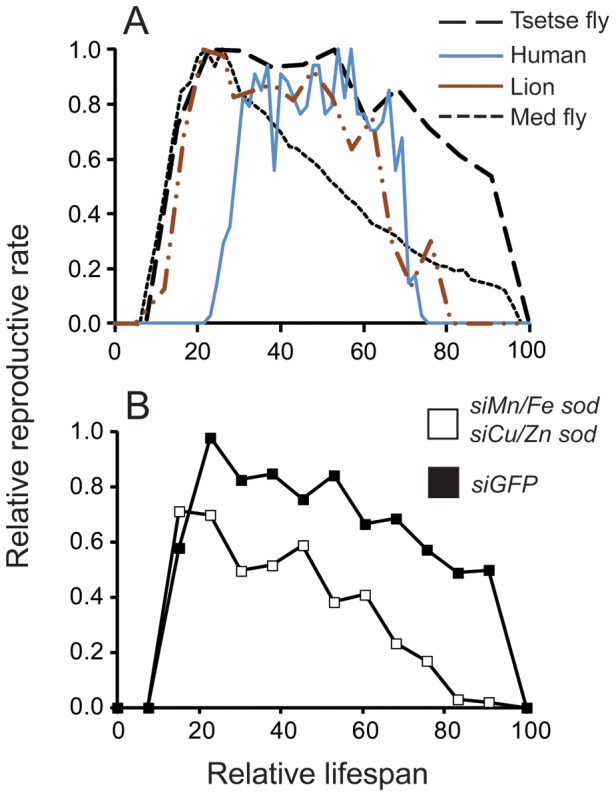
Age-related fecundity patterns in various species in comparison to tsetse flies. A. Medfly, (eggs/day) [46], [47], human (traditional Ache population, progeny/year) [101], lions (progeny/year) [102], *Drosophila melanogaster* (eggs/day) [103] and tsetse fly (this study, [33]). B. Age-related fecundity patterns in tsetse fly after knockdown *Mn/Fe sod* and *Cu/Zn sod*. Mean ± SE for three groups of 15 flies.

We compared data on reproductive rate relative to lifespan between tsetse and other organisms to examine how reproduction/aging dynamics of this fly relates to that of other organisms (Figure 6). In general, there is a period of time before an organism becomes reproductively active (Figure 6a). A decline in reproductive capacity usually occurs as organisms age. This typically happens gradually in insects such as med fly and *Drosophila* (Fig. 6a; [45]–[48]), but more rapidly after a critical age in the case of mammals, lions, and most other K-strategists. This rapid loss of fecundity represents declining reproductive fitness with age, or reproductive senescence (Figure 6a). Tsetse flies represent a drastic deviation from other animals in that they maintain their fecundity late in life (Figure 6a). Suppression of the two *sod* genes during lactation, birth and involution, however reduce tsetse's fecundity, making it more comparable with the fecundity parameters of other organisms. This is marked by a decline in the fecundity observed with age, and by a period of reproductive senescence late in life (Figure 6b). These results indicate that the increased expression of AOEs during lactation, birth and involution are critical for the prevention of reproductive decline and senescence.

Tsetse flies unique ability to maintain a high level of fecundity late in life is comparable to that of the naked mole rat, a rodent known for extreme longevity and high fecundity in old age [34]. Tsetse flies also live longer than other related insects and have no apparent decline in fecundity [34]. The lifespan (longevity) of both tsetse and the naked mole rat does not seem to be impacted by the act of reproduction. Our findings in tsetse implicate AOE expression at critical periods during each pregnancy cycle as a necessary mechanism that allows tsetse to maintain reproductive fitness and minimizes end of life reproductive senescence.

### Pregnancy Stress can be Experimentally Induced by Exogenous H_2_O_2_ Treatment Followed by *sod* Knockdown

**Figure 7:**
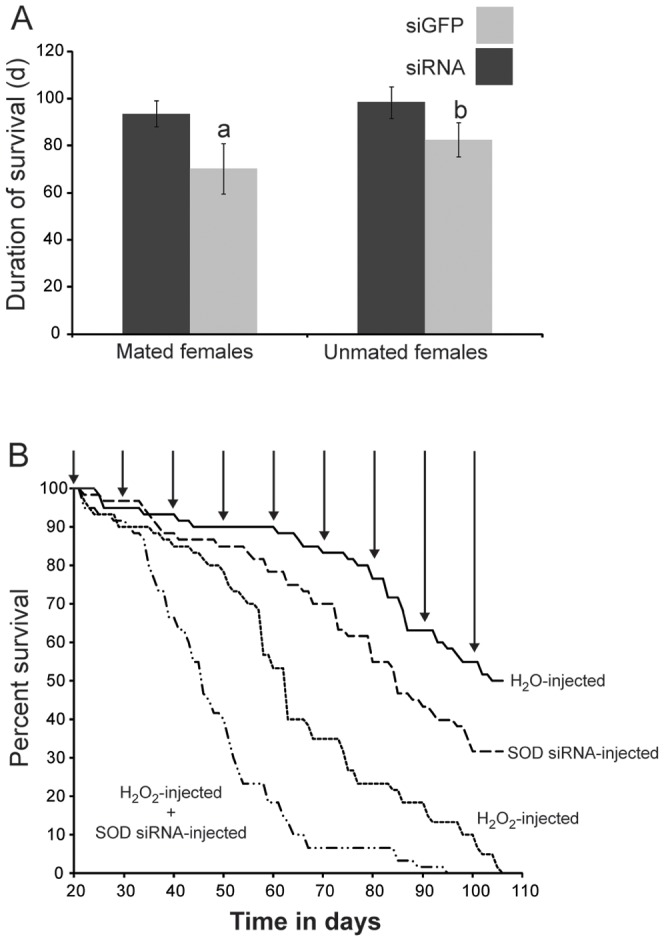
Survival of pregnant and virgin females following *sod* knockdown and exogenous treatment with H_2_O_2_. A. Longevity following *sod* knockdown in mated and unmated females. Mean ± SE of 15 flies. B. Survival of groups of virgin flies to mimic subjected to one of four treatments: H_2_O or H_2_O_2_ injections at intervals during the peak of lactation that matched those of tsetse fly pregnancy, *Mn/Fe* and *Cu/Zn sod* during the first three pregnancy cycles and *Mn/Fe* and *Cu/Zn sod* during the first three pregnancy cycles along with H_2_O_2_ at intervals that match those of pregnancy. Survival data was measured using a Kaplan-Meier plot along with a log rank test. Arrows indicate treatment with H_2_O or H_2_O_2_.

We noted a decrease in overall longevity upon *sod* knockdown, with the effect being more prominent in reproductively active flies (Figure 7a). Next we investigated whether increased lactation associated OS may also be responsible for the noted decrease in tsetse's longevity. We used exogenous H_2_O_2_ treatment to induce OS at critical times, mimicking the pregnancy-induced responses in age matched virgin females for comparison with pregnant but untreated counterparts. We injected virgin females with H_2_O_2_ at 10 day intervals, which correspond to the beginning of each pregnancy cycle starting on day 20 when females typically carry their first late stage third instar larva prior to parturition. These experiments were to specifically address if the bouts of oxidative stress associated with reproduction impact fly longevity without other physiological changes associated with lactation and birth, specifically after the reduction of the AOE response. We followed these flies for effects on longevity until death. Injection of virgin females with exogenous H_2_O_2_ resulted in a substantial decline in longevity with 50% mortality occurring nearly 50 days earlier than in the control virgin female group that received H_2_O injections (Figure 7b). We next treated a group of virgin females with si*Mn/Fe* and si*Cu/Zn sod*, and found a small but significant reduction in their longevity when compared to control groups similarly injected with H_2_O and siGFP, respectively (Figure 7b). However, injection with H_2_O_2_ after knockdown of *Mn/Fe* and *Cu/Zn sod* resulted in a drastic reduction in lifespan, which was not observed in either the siGFP or the H_2_O_2_ treatment groups (Figure 7b). These results indicate that OS, similar to that which occurs during lactation and parturition in tsetse, is particularly more detrimental to lifespan at times when SOD levels are low.

### The SOD Response is Essential for Tsetse Population Sustainability

**Figure 8:**
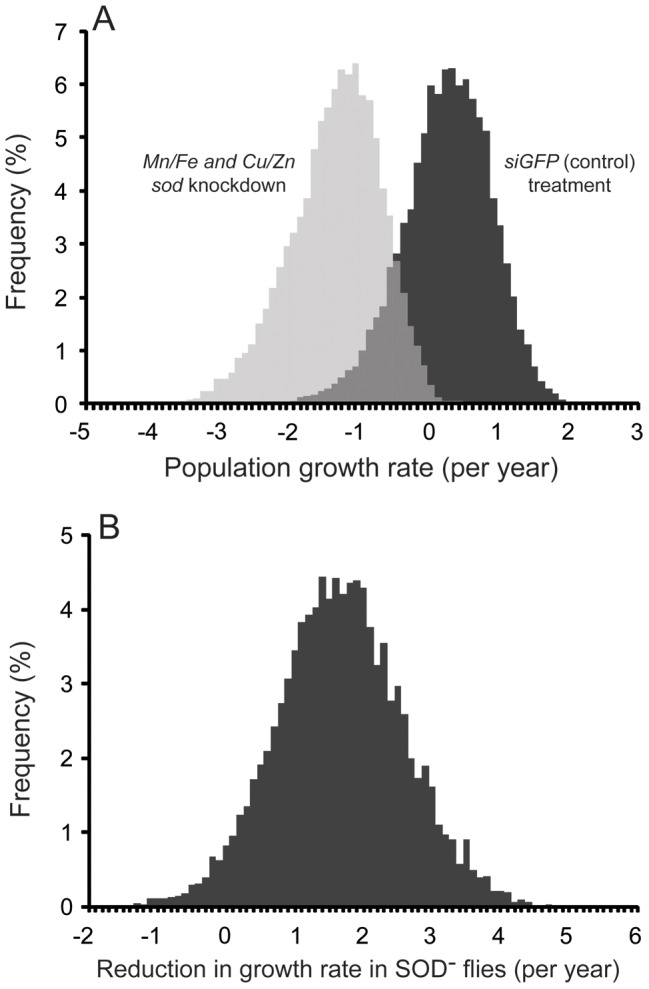
Population modeling following *sod* knockdown. A. Frequency of growth rate. B. Reduction in growth rate between *sod* knockdown and control (*siGFP*). Results represent 10,000 simulated replicates.

We performed population modeling to determine how suppression of the lactation-associated SOD response might alter population growth dynamics using our data. Based on the modeling analysis, *siGFP* treated control flies had a probability of positive growth of 62.8% (Figure 8a). When flies were treated with *siMn/Fe sod* and *si Cu/Zn sod* double knockdown, we noted a negative impact on population growth, with only a 0.3% probability of a positive growth rate, the level necessary to support replacement of flies dying in the population (Figure 8a). Direct comparison of the growth rates between control and knockdown flies revealed an absolute difference in growth rate of 1.61 per year (95% CI = −0.214, 3.473, p-value for difference >0 = 4.06%; Figure 8b). The absolute values correspond to a decline in population size of 80.0% [95% confidence interval = −23.9%, 96.9%] after one year in the absence of an adequate SOD response during lactation. The modeling results indicate that the reproductive-associated antioxidant response is likely critical to maintain tsetse populations in the wild.

## Discussion

**Figure 9:**
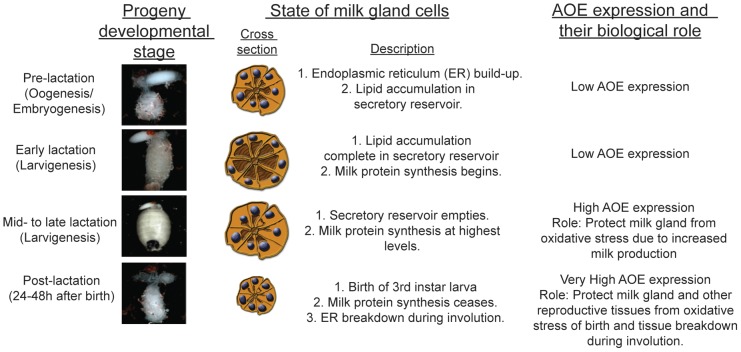
Summary for the role of oxidative stress and antioxidant enzyme expression during tsetse fly reproduction. Developmental images adapted from Benoit et al. [104]. Cross sections adapted from Ma and Denlinger [28], Hecker and Moloo [105] and Yang et al. [41].

In this study we used the viviparous tsetse fly to investigate the role of oxidative stress and antioxidant enzymes in reproductive senescence and female longevity. Tsetse females remain fertile for much of their adult-life, and once mated undergo multiple pregnancies (8–10) with no evidence of significant reproductive senescence. During this pregnancy period, they lactate to nurture their progeny to full term in their uterus by milk secretions produced from the milk gland organ. It appears that tsetse females can successfully manage reproduction-associated OS during the lactation, parturition and involution periods of the reproductive cycle. We found high levels of expression of the antioxidant pathway genes (*Cu/Zn sod*, *Mn/Fe sod* and *catalase*) late in pregnancy, and immediately after birth in reproduction associated tissues (milk gland/fat body and uterus). This increase in AOE appears to be important to maintain homeostasis with each subsequent pregnancy cycle, to prevent direct damage to the milk gland, and prevent interference with other underlying mechanisms necessary for lactation. Knockdown of the SOD response during lactation reduces milk protein generation in the subsequent reproductive cycles, results in reproductive senescence in older flies, and reduces longevity, particularly in reproductively active females. These results suggest that the spatially and temporally regulated AOE responses are a critical mechanism that protects the female reproductive system from OS over multiple cycles of lactation, parturition and involution. Population modeling predicts that if tsetse females lack this lactation associated AOE response, natural population growth would decline by 80% within the year. We summarize the role of AOE expression during tsetse reproduction in Figure 9.

Similar to mammalian lactation, tsetse females nourish their progeny via secretion of milk from the milk gland organ [37], [39], [40]. Tsetse milk composition and production shares several similarities with mammalian systems. Mammalian systems have highly specialized lactating cells that cycle through periods of high activity during lactation to low/no activity following involution during the dry period [49], [50]. The protein composition of tsetse and mammalian milk also show functional analogy between the two systems [51], [52]. Comparable protein types include a lipocalin (MGP1 in tsetse versus β-lactoglobulin in mammals [39], [53]–[56]), an iron-binding protein (Transferrin in tsetse versus Lactoferrin in mammals [38], [57]), sphingomyelinase present either in milk or in the gut contents of nursing progeny [37], [58]–[60] as well as immune proteins (peptidoglycan recognition protein PGRP-LB and UBASH3A in tsetse versus multiple mammalian immune proteins [40], [51], [54], [61], ). The lipid content of the milk secretions transferred to the developing offspring during lactation is also similar in both systems, both in composition and concentration [29], [31], [63]. In tsetse, the obligate symbiont *Wigglesworthia*, which provides critical nutrients for maintaining fecundity and for immune maturation, is transferred maternally in milk secretions [53], . Symbiotic bacteria in maternal milk secretions are also found in mammalian systems, and are thought to function as probiotics [64]–[65]. Lastly, tsetse and mammals have an expanded family of lactation-specific proteins (MGP 2–10 family in tsetse versus casein family in mammals [40], [66], [67]). The convergence between these disparate systems suggests that tsetse flies have developed alternative yet functionally similar solutions to the problem of providing immature offspring with essential nutritional requirements.

The critical role of OS in relation to reproduction and progeny nourishment has been documented in many organisms, including *Drosophila* [68], [69], birds [70], [71], mammals [2], [72], [73] and blood feeding insects, such as sand flies and mosquitoes [8], [9]. In terms of milk production, dairy cows express increased OS markers during lactation and in the subsequent period of involution [74]. However, increased OS during reproduction has not been documented for all organisms. Female house mice and bank voles do not show increased markers for OS during reproduction [75], [76]. The lack of oxidative damage is not surprising as reproductive evolution has likely equipped animals with the ability to mitigate immediate and long-term damage associated with reproduction to maintain fecundity [77]. Similarly, in tsetse flies OS damage does not increase over the course of reproduction, and does not differ between fertile and infertile individuals. This is most likely due to increased AOE response mediated by the expression of two SOD genes and a catalase gene late in intrauterine larval development, at parturition and during the subsequent period of involution that likely prevents excess ROS. Indeed, when RNA interference was utilized to reduce AOE genes during lactation, we observed a significant increase in oxidative damage during the subsequent periods of lactation and pregnancy. This damage resulted in impaired progeny output due to direct milk gland dysfunction or other mechanisms by which milk protein synthesis is impaired (i.e. reduction of available nutrients for milk protein synthesis). These results support the hypothesis that tsetse flies are adapted to mitigate the OS generated by the high energy demands of their intrauterine progeny by utilizing AOE pathways.

One of the most interesting findings from this study is there is no or minimal decline in fecundity as tsetse flies age. A few organisms, such as the naked mole rat [34], have high levels of progeny output during the later periods of life. For our studies, it is important to mention that our flies were held under laboratory conditions with regular access to meals throughout the course of the study. Although these conditions would seem to be optimal, flies do experience mechanical damage to their wings and bodies from housing within cages, suggesting that these flies likely experience stress throughout their lifetimes. However, colony conditions may be more stable than field conditions, thus OS is likely to be one of the many contributing factors involved in tsetse reproductive senescence. As an example, tsetse flies maintain tight control of lipid levels during the transition between lactation and dry periods [29], [78]. These lipid reserves are critical for providing fat for incorporation into the milk and their breakdown generates amino acids necessary for milk protein generation [29]–[32]. If aging prevented lipid accumulation between periods of lactation,, potentially through dysregulation of insulin or juvenile hormone signaling that have been shown to regulate lipid homeostasis [78], flies may experience reproductive senescence due to the inability of older mothers to produce milk at adequate levels. Thus, there may be other factors beyond oxidative stress that could contribute to reproductive senescence in field flies. Even so, our study demonstrates that tsetse females lacking an oxidative stress response during lactation and birth have a discernible period of reproductive senescence and will be unable to maintain reproduction above replacement levels based on population modeling.

In relation to aging, mated and unmated tsetse females have the same lifespan (this study; [33]). This indicates that under laboratory conditions, there is no trade-off between fecundity and life span. This finding supports the discoveries reported in recent studies across multiple animal systems ranging from insects to mammals, which show that reproduction apparently has little to no effect on longevity [45], [48], [72], [79]. Other studies have shown the fitness consequences due to reproduction such as impaired immunity and reduced resistance to environmental stress [25], [72], [80]–[82]. Our finding in tsetse shows that fecundity has a substantial cost on longevity when the AOE response is suppressed during reproduction. Recent studies have questioned the role of the OS response in relation to aging, since little to no change in lifespan was shown to occur after knockdown of *sod* genes [18], [83], and overexpression of AOEs did not appear to extend lifespan in multiple model species [11], [83]. Many of these studies however have utilized optimal rearing conditions, and it has been suggested that under suboptimal conditions OS could play a more important role in relation to aging [13]. It is possible that the observed impact on longevity in tsetse may not be due solely to oxidative damage, but may include other factors as well. A recent theory that has garnered support is the hyperfunction theory of aging, proposed by Williams [84] and Hamilton [85] with mechanisms focused on by Blagosklonny [86]–[89]. This theory argues that unregulated processes associated with early-life fitness, such as growth and reproduction, can be damaging later in life. These uncontrolled processes can lead to negative-age related phenotypes and death. We have observed increased lipid levels in older flies, specifically in flies with a reduced ability to respond to OS during lactation. The inability to maintain lipid homeostasis suggests possible disruptions in the target of rapamycin (TOR), insulin signaling, or alternative nutrient sensing pathways, which is a major hallmark of aging [90]. Along with improper nutrient signaling, other hallmarks of aging, such as mitochondrial dysfunction and loss of proteostasis, may be altered in flies without their normal response to OS during lactation. Additional studies will be necessary to pinpoint the underlying pathologies that result from interference with the AOE response during tsetse lactation. Even so, our study suggests that reproductive-associated OS will lead to decreased longevity if the mechanisms for its suppression are impaired.

### Summary

In this study, we show that the AOE response is critical for tsetse flies to manage OS generated during pregnancy and to maintain fecundity, particularly late in life. In the absence of the AOE response, there is a precipitous decline in fecundity, which is due, at least in part, to the inability of tsetse mothers to synthesize the milk nutrients required for progeny development. Population modeling reveals that in the absence of a well-regulated AOE response, the population growth rate will fall below replacement levels. These results suggest that the AOE response is likely a critical aspect that functions to compensate for the OS resulting from the massive metabolic demands generated by lactation in tsetse. In relation to organismal reproduction and senescence, it is likely that OS occurs during reproduction in most organisms, but females may have adapted to prevent the accumulation of oxidative damage during this period to allow for normal reproductive output as aging occurs.

## Materials and Methods

### Flies and antioxidant genes

Laboratory colonies of *Glossina morsitans morsitans*, established from a population collected in Zimbabwe, are utilized at Yale University for at least 20 years. Flies are maintained at 25°C and 50–60% RH using an artificial membrane feeding system [91]. Females were mated 3–5 d after emergence and collected according to developmental markers based on oocyte, embryo and larva presence [26], [39]. Multiple antioxidant genes have been identified from tsetse flies based on previous studies (Table S1; [92]). Flies were stored in groups of 5 throughout our experiments for uniformity. We have provided a diagram of the experimental set-up to more easily interpret the results (Figure S5).

### RNA and protein extraction

Temporal samples were acquired from pregnant flies with developing progeny removed so that transcript levels reflect only the changes occurring within the mother. Spatial samples were acquired from pregnant females with a 3^rd^ instar larvae present within the uterus. Each sample was collected 24 h after blood feeding to remove effects of digestion. RNA and protein were extracted from whole flies and tissues using Trizol reagent according to the manufacturer's protocol (Invitrogen, Carlsbad, CA). cDNA was synthesized from 1 µg of total RNA using Superscript III reverse transcriptase kit (Invitrogen) based on the manufacturer's protocol, with the exception that cDNA synthesis was extended to 60 min. RNA and cDNA were stored at −70°C until use. Protein was stored in protein pellet solubilization buffer (8M urea, 3M thiourea, 1% dithiothreitol and 4% CHAPS) at −20°C.

### Transcript expression levels

The PCR amplification conditions were 95°C for 3 min, thirty cycles of 30 s at 95°C, 52 or 56°C for 1 min, and 1 min at 70°C in a Bio-Rad DNA Engine Peltier Thermocycler (Hercules, CA) with gene-specific primer sets. PCR products were purified and the amplified regions were validated by sequencing at the DNA Analysis Facility at Yale University. Levels of antioxidant and lactation-specific gene expression were determined by qPCR utilizing the iCycler iQ real-time PCR detection system (Bio-Rad, Hercules, CA) using gene specific primers (Table S1). The data were obtained in triplicate samples and were normalized to tsetse *tubulin* (*tub*, DQ377071.1) expression levels and analyzed with software version 3.1 (Bio-Rad). Transcript levels were assessed throughout the course of pregnancy for antioxidant genes, following knockdown of specific genes for validation and to determine if transcript levels of milk proteins were alters after perturbation of lactation-associated antioxidant responses (Figure S5).

### RNA interference of antioxidant genes

Short interfering RNAs (siRNA) were synthesized commercially (IDT, Coralville, IA) and consist of two duplex sequences for *Cu/Zn sod* (CCGACGUGACGUAUCUCA and GUACUGAUGAGAUACGUC) and *Mn/Fe sod* (AAUUGGAGCCAAAGCACC and CGACUAUGGUGCUUUGGC). Control siRNAs were designed against green fluorescent protein (GFP; GAUGCCAUUCUUUGGUUUGUCUCCCAU and CUUGACUUCAGCACGUGUCUUGUAGUU). The concentration was determined by spectrophotometer and adjusted to 1 µg/µl. Each fly in the first treatment group was injected with 1.5 µl siRNA13d after emergence (single treatment) and flies in a second treatment group were injected again at 23 d (two treatments). This treatment procedure allowed for interference of the lactation-associated antioxidant response. For validation, transcript expression levels were determined by qPCR, antioxidant capacity and H_2_O_2_ tolerance was assessed 5–6 d after siRNA injections. Fecundity of the flies was measured over multiple gonotrophic cycles and displayed as the duration of pregnancy and the number of progeny generated per females during each gonotrophic cycle

### Western blot analysis of tsetse milk proteins

Equal volumes of protein from three flies were combined for each time point, and analyzed by standard western blot protocol [39]. Antisera against Tubulin (Tub), Milk Gland Protein (MGP1) and Transferrin (Trf) were previously generated against recombinant tsetse proteins [38], [39]. Analysis of Tubulin and MGP were performed utilizing the protein equivalent of 1/100th of a fly per well. Blots were blocked overnight in PBS, 3% BSA and 0.5% Tween 20 (blocking buffer). Tub and MGP1/Trf antisera were utilized at 1∶5,000 and 1∶20,000, respectively [38], [39]. Signals were visualized with Supersignal West Pico Substrate (Pierce, Wobrun, MA) on an Image Station 2000R (Kodak, New Haven, CT). Western blot analysis was conducted for flies during the 3^rd^ gonotrophic cycle after reduction of the AOE response in the previous two reproductive cycles (Figure S5).

### In situ hybridization

Milk gland tubules together with fat body were collected from female flies carrying third instar larva and placed into Carnoy's fixative for a five day fixation period [53]. Digoxigenin-labeled RNA probes were generated using the MAXIscript T7 transcription kit following manufacturer's protocol (Ambion, Austin, TX) using a primer set with a T7 primer (Table S1) [52]. Antibody solutions were made featuring anti-Digoxigenin-rhodamine Fab fragments for FISH probe detection (1∶200 dilution) (Roche) and rabbit anti-GmmMGP (1∶2500) antibodies [39], [53]. Alexa Fluor 488 goat anti-rabbit IgG (Invitrogen) at a dilution of 1∶500 was added as a secondary antibody for immunohistochemistry [53]. Slides were mounted in VECTASHIELD Mounting Medium with DAPI (Vector laboratories Inc. Burlingame, CA). Samples were observed using a Zeiss Axioskop2 microscope (Zeiss, Thornwood, NY) equipped with a fluorescent filter. Samples were viewed and imaged at 400× magnification. Images were captured using an Infinity1 USB 2.0 camera and software (Lumenera Corporation, Ottawa, Ontario, Canada).

### Trolox-equivalent antioxidant capacity (TEAC) assay

The TEAC assay was conducted according to Re et al [93], with modification by Lopez-Martinez et al. [88]. Female flies and dissected tissues were frozen in liquid nitrogen and stored at −70°C until analysis. TEAC was measured using 2,2′-azino-bis-(3- ethylbenzothiazoline-6-sulfonic acid) (ABTS) radical cation decolorization assay. Samples were compared to a Trolox standard curve [0–150 µmol l^−1^ (ml^−1^)] and results were expressed at Trolox equivalents per me soluble protein. Three pools of three samples were homogenized in PBS and centrifuged at 5000 g for 5 min at 4°C. The fly homogenate was diluted to a concentration of 2 mg protein ml^−1^. The ABTS (7 mmol l^−1^ in 2.45 mmol l^−1^ potassium persulfate; Sigma-Aldrich, St Louis, MO, USA) was prepared before the assay and allowed to equilibrate in the dark at 25°C overnight. The protein samples and Trolox standard were combined with ABTS and incubated for 10 min at 25°C. Antioxidant capacity was measured at 734 nm on Synergy HT Multi-Mode Microplate Reader (BioTek). TEAC levels were assessed through the tsetse pregnancy cycle from females after removal of an embryo, 1^st^ instar, 2^nd^ instar and 3^rd^ instar larva from the uterus along with those immediately birth (no progeny in the uterus). In addition, TEAC levels were assessed following knockdown of *Cu/Zn sod* and *Mn/Fe sod* to determine if their knockdown impaired antioxidant levels.

### Lipid peroxidation assay

Lipid peroxidation was measured by the amount of malondialdehyde (MDA), the main aldehyde product of lipid peroxidation, utilizing the thiobarbituric acid reactive substances test (TBARS). This assay was adapted from those previously described [94]–[97]. Individual flies, or tissues dissected from three individuals were pooled and homogenized in RIPA buffer with EDTA, respectively. The samples were split into two aliquots: one was used to determine the protein concentration as standardization and the second was treated with 10% trichloroacetic acid (TCA) then incubated in ice to precipitate proteins. Following TCA treatment, the sample was centrifuged for 2200 g for 15 min at 4°C. The supernatant was combined with 0.6% (w/v) thiobarbituric acid solution and heated at 95°C for 1 h. The samples were then allowed to cool to 25°C on ice and centrifuged (2200 g for 5 min) prior to absorbance measurement at 532 nm. MDA quantification was determined using an eight point MDA standard curve [0–50 µmol l^−1^] standardized in relation to total protein. MDA levels were assessed through the tsetse pregnancy cycle from females after removal of an embryo, 1^st^ instar, 2^nd^ instar and 3^rd^ instar larva from the uterus along with those immediately birth (no progeny in the uterus; Figure S5). In addition, MDA levels were assessed in the third reproductive cycle following knockdown of *Cu/Zn sod* and *Mn/Fe sod* in the first two gonotrophic cycles to determine if their knockdown resulted in increased MDA levels.

### Protein carbonyl assay

Protein oxidation was measured according to Levine et al. [98] with modifications by Lopez-Martinez et al. [95]. Whole flies and tissues were homogenized in a 5% sulfosalicylic acid. Excess amount of 2,4-dinitrophenylhydrazine (DNPH, Sigma) was utilized to extract carbonyls, the carbonyls were precipitated with TCA and multiple washes with ethanol∶ethyl acetate solutions was utilized to remove excess DNPH. The resulting proteins were diluted in 6M guanidine hydrochloride. Sample absorbance was measured at 370 nm and compared to a BSA standard (nine points, 0.2 mg/ml) curve. Data are presented as nmol mg^−1^ soluble protein. Protein carbonyl levels were assessed through the tsetse pregnancy cycle from females after removal of an embryo, 1^st^ instar, 2^nd^ instar and 3^rd^ instar larva from the uterus along with those immediately birth (no progeny in the uterus; Figure S5). In addition, protein carbonyl levels were assessed in the third reproductive cycle following knockdown of *Cu/Zn sod* and *Mn/Fe sod* in the first two gonotrophic cycles to determine if their knockdown resulted in increased protein carbonyl levels.

### Effect of exogenous OS on nonreproductive (virgin) flies

For testing the periodic OS, unmated female flies were utilized under four separate treatment groups (Figure S5). The first treatment group was injected with 2 µl of 0.5% H_2_O_2_ at intervals of pregnancy (20, 30 and 40 d) during the first three reproductive cycles. The second treatment group was injected with water control, *Mn/Fe sod* and *Cu/Zn sod* siRNA (200 ng/µl) to determine the effect of SOD knockdown on survival, respectively. The third treatment group received *Mn/Fe sod* and *Cu/Zn sod* siRNA (200 ng/µl) along with 0.5% H_2_O_2_ to determine the effect of increased OS after SOD knockout, respectively. Flies were held under standard colony rearing protocols and monitored until 100% mortality was reached. An outline of the experimental design is provided in Figure S5.

### Population modeling following reduction of SOD response

To estimate the impact of reproductive-associated antioxidant response at the population scale, we built a simple mathematical model of a tsetse population growth. The model population for impaired reproductive-associated antioxidant response was parametrized with data from the si*Mn/FE* and si*Cu/Zn sod* treatments, while the non-impaired model population was parametrized with data from the control treatment, siGFP treatment (Table S3). For both model populations, we assumed normal, non-antioxidant-impaired mortality. We calculated the mean and standard deviation of fecundity and gonotrophic-cycle length for the control group and for the combined si*Mn/FE* and si*Cu/Zn sod* treatment group for 12 gonotrophic cycles (Table S3). For each gonotrophic cycle and each treatment group, we modeled fecundity *F_jk_* as a beta random variable with parameters chosen to match the mean and standard deviation of the data. Similarly, we modeled gonotrophic-cycle length *t_jk_* as a log normal random variable with parameters chosen to match the mean and standard deviation of the data for each gonotrophic cycle and each treatment group.

Given values of fecundity and gonotrophic cycle length, number of female offspring produced by a single female tsetse over its lifetime is *R_j_* = *p* ∑ *F_jk_ S_jk_* where *p* is the probability than a deposited pupa is female, which we took to be 55% [99] and *S_jk_* is the survival, the probability of surviving to gonotrophic cycle *k*. We modeled survival as *S_jk_* = *S*_pupa_ *s^T_jk_^*, where *S*_pupa_ is the probability that a deposited pupa survives to emerge as an adult, which we took to be a conservative number at 85% [99], [100]; *s* is the probability of surviving each adult day, which we took to be 98% [99], [100], and *T_jk_* = ∑ *t_jk_* is the number of days from emergence until the end of gonotrophic cycle *k*. The population growth rate is then *r_j_ = R_j_D*, with the generation time defined to be *D = D*_pupa_+*D*_adult_ is the mean duration of the pupal stage, which we took to be 31.4 days [100]; and *D*_adult_ = −(log *s*)*^−1^* is the mean adult lifespan. We calculated the population growth rate *r_j_* for each treatment group and the difference in growth rate between the two treatment groups, *r*_1_−*r_2_*, for 10,000 samples of our model.

### Statistical analysis

Results were compared utilizing JMP or SAS statistical software programs (Cary, North Carolina, USA). Mean differences utilized between treatments were compared with one-way or two-way ANOVA with a Bonferroni correction followed by Tukey's post-hoc test. A Kaplan-Meier analysis was utilized to measure survival differences following treatment with H_2_O_2_.

## Supporting Information

Figure S1 **Antioxidant gene and activity levels in specific tissues before, during and after lactation.** A, B and C. Transcript levels for *Mn/Fe superoxide dismutase* (*Mn/Fe sod*), *Cu/Zn sod* and *catalase*, respectively Each point represents the mean ± SE of four measurements. D. Antioxidant activity determined as Trolox-equivalent assay (µmol l^−1^ mg^−1^ protein). Each sample represents the mean ± SE of three samples.
(TIF)

Figure S2 **Fluorescent** ***in situ*** **hybridization (FISH) analysis.** Red for *Mn/FE sod* (A) and *Cu/Zn sod* (B) green for milk gland protein (MGP) immunohistochemistry. DAPI staining of nuclei in blue, is shown in a cross section of milk gland tubules. 1 = milk gland lumen; 2 = nuclei; 3 = secretory reservoir. Negative controls not treated with Digoxigenin-labeled antisense RNA probes displayed no signal.
(TIF)

Figure S3 **RNA interference of** ***Mn/Fe sod*** **and** ***Cu/Zn sod*** **.** A. Transcript levels. Mean ± SE of three samples. B. Antioxidant activity. Mean ± SE of four samples. C. Resistance to H_2_O_2_ injection. Mean ± SE of 15 flies.
(TIF)

Figure S4 **Reduction in milk gland protein levels after SOD gene knockdown.** Tubulin was utilized as an internal control. Relative protein levels were determined with densitometry through the utilization of ImageJ. Mean ± SE of three blots.
(TIF)

Figure S5 **Diagram outlining experimental design.**
(TIF)

Table S1 **Quantitative PCR primer information utilized in this study.**
(XLS)

Table S2 **qPCR expression of multiple oxidative stress genes through tsetse pregnancy.**
(XLSX)

Table S3 **Gonotrophic cycle length and fecundity by cycle number for control and si** ***Mn/Fe*** **and si** ***Cu/Zn sod*** **treatment groups.** Cycle length is the duration of the gonotrophic cycle in days. Fecundity is probability that pupa was deposited during the cycle.
(XLSX)
