# Supplementary figures and images for: Amelioration of Reproduction-Associated Oxidative Stress in a Viviparous Insect Is Critical to Prevent Reproductive Senescence
Source: PLoS One. 2014 Apr 24;9(4):e87554. doi: 10.1371/journal.pone.0087554 (PMC3998933; doi:10.1371/journal.pone.0087554)

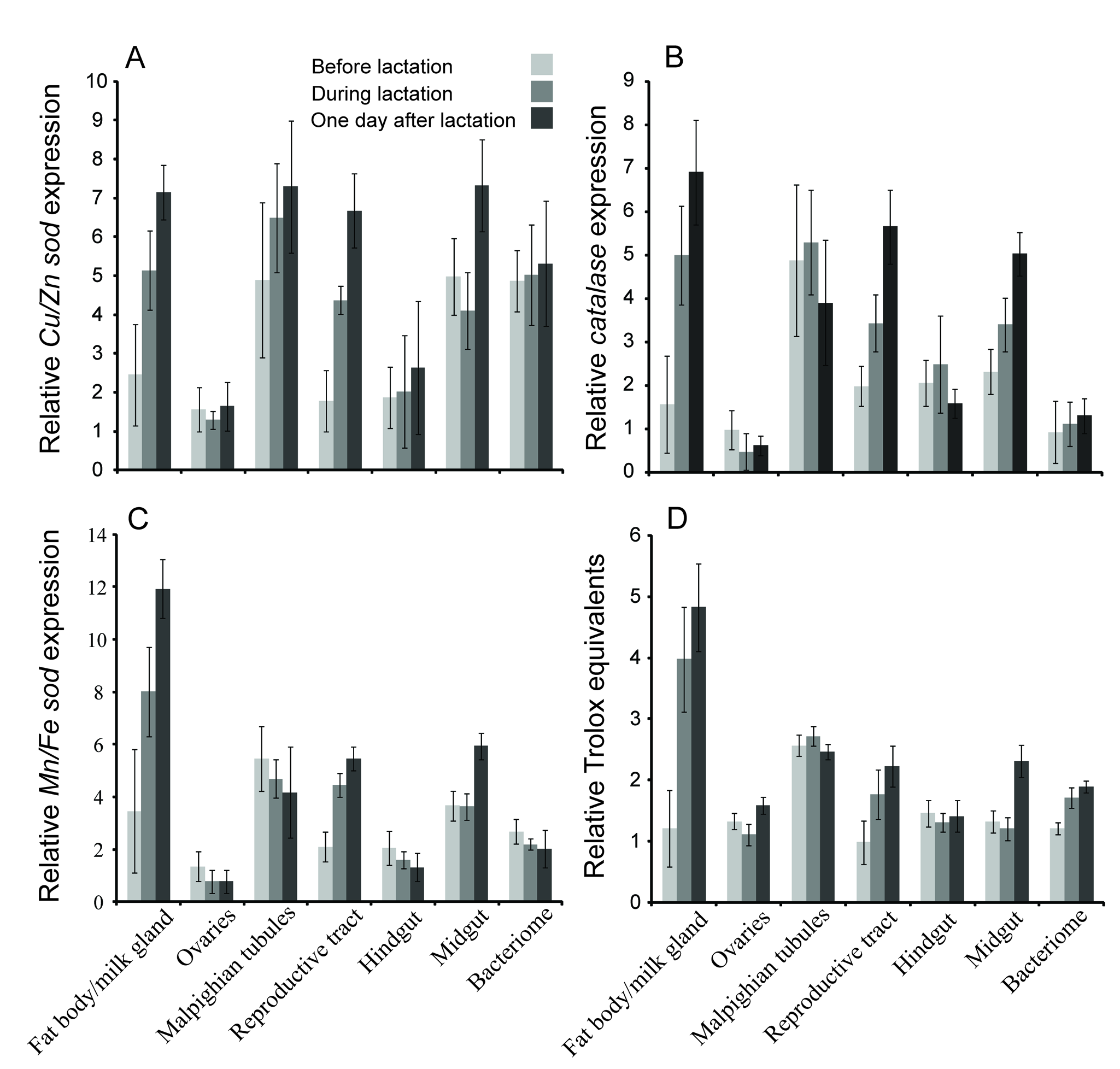

Supplement: Figure S1 — Antioxidant gene and activity levels in specific tissues before, during and after lactation. A, B and C. Transcript levels for Mn/Fe superoxide dismutase (Mn/Fe sod), Cu/Zn sod and catalase, respectively Each point represents the mean ± SE of four measurements. D. Antioxidant activity determined as Trolox-equivalent assay (µmol l−1 mg−1 protein). Each sample represents the mean ± SE of three samples. (TIF) [file pone.0087554.s001.tif]

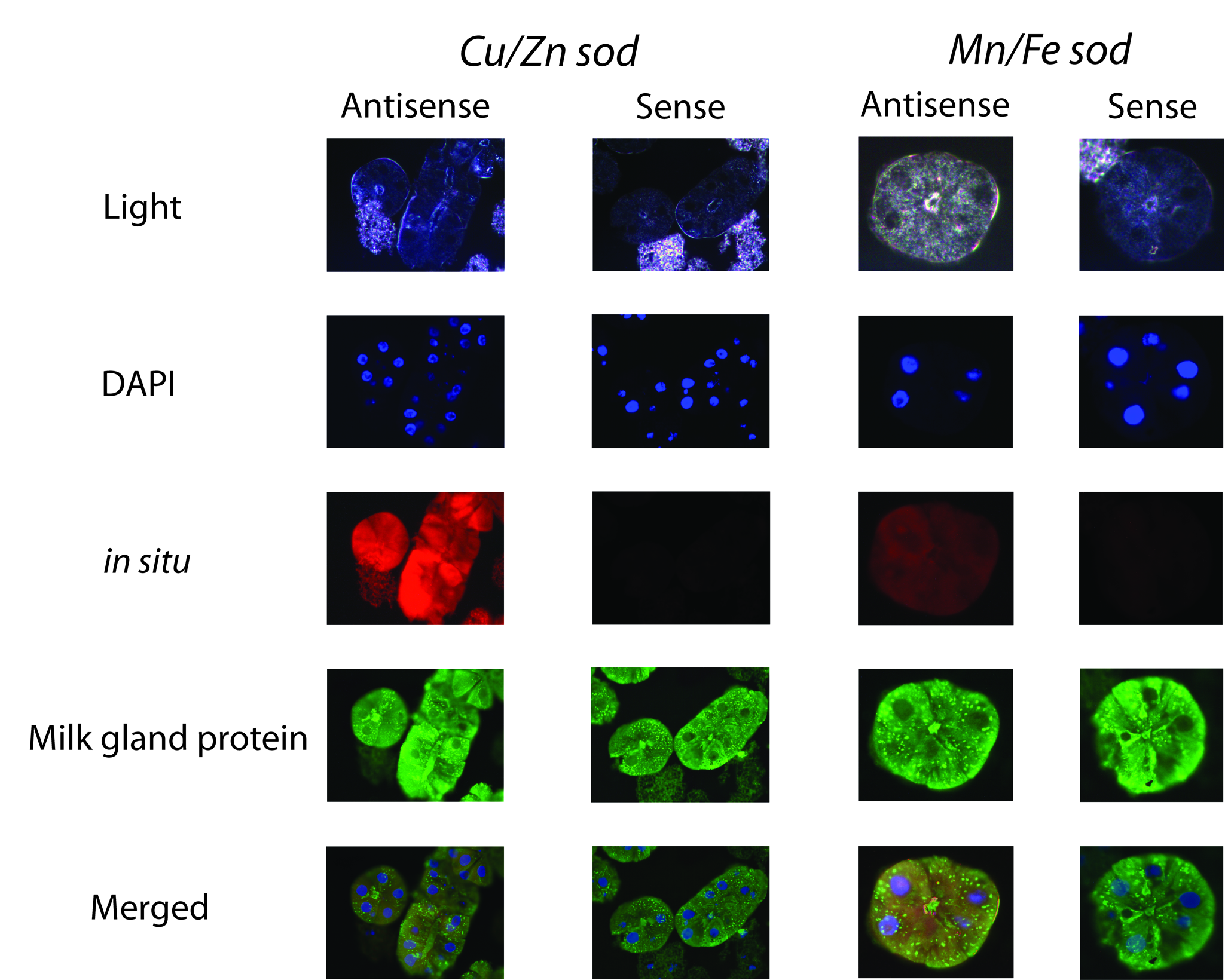

Supplement: Figure S2 — Fluorescent in situ hybridization (FISH) analysis. Red for Mn/FE sod (A) and Cu/Zn sod (B) green for milk gland protein (MGP) immunohistochemistry. DAPI staining of nuclei in blue, is shown in a cross section of milk gland tubules. 1 = milk gland lumen; 2 = nuclei; 3 = secretory reservoir. Negative controls not treated with Digoxigenin-labeled antisense RNA probes displayed no signal. (TIF) [file pone.0087554.s002.tif]

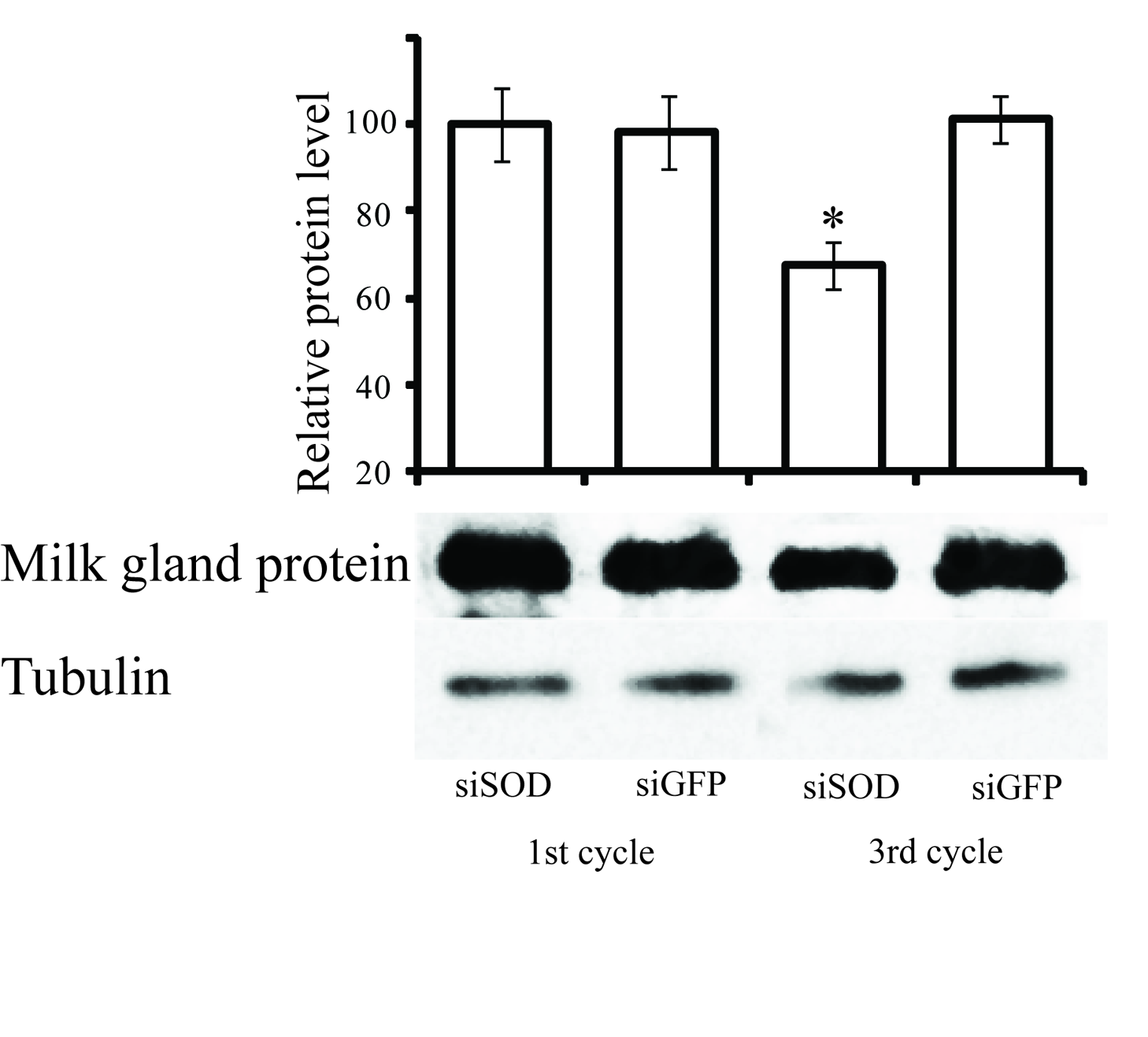

Supplement: Figure S3 — RNA interference of Mn/Fe sod and Cu/Zn sod . A. Transcript levels. Mean ± SE of three samples. B. Antioxidant activity. Mean ± SE of four samples. C. Resistance to H2O2 injection. Mean ± SE of 15 flies. (TIF) [file pone.0087554.s003.tif]

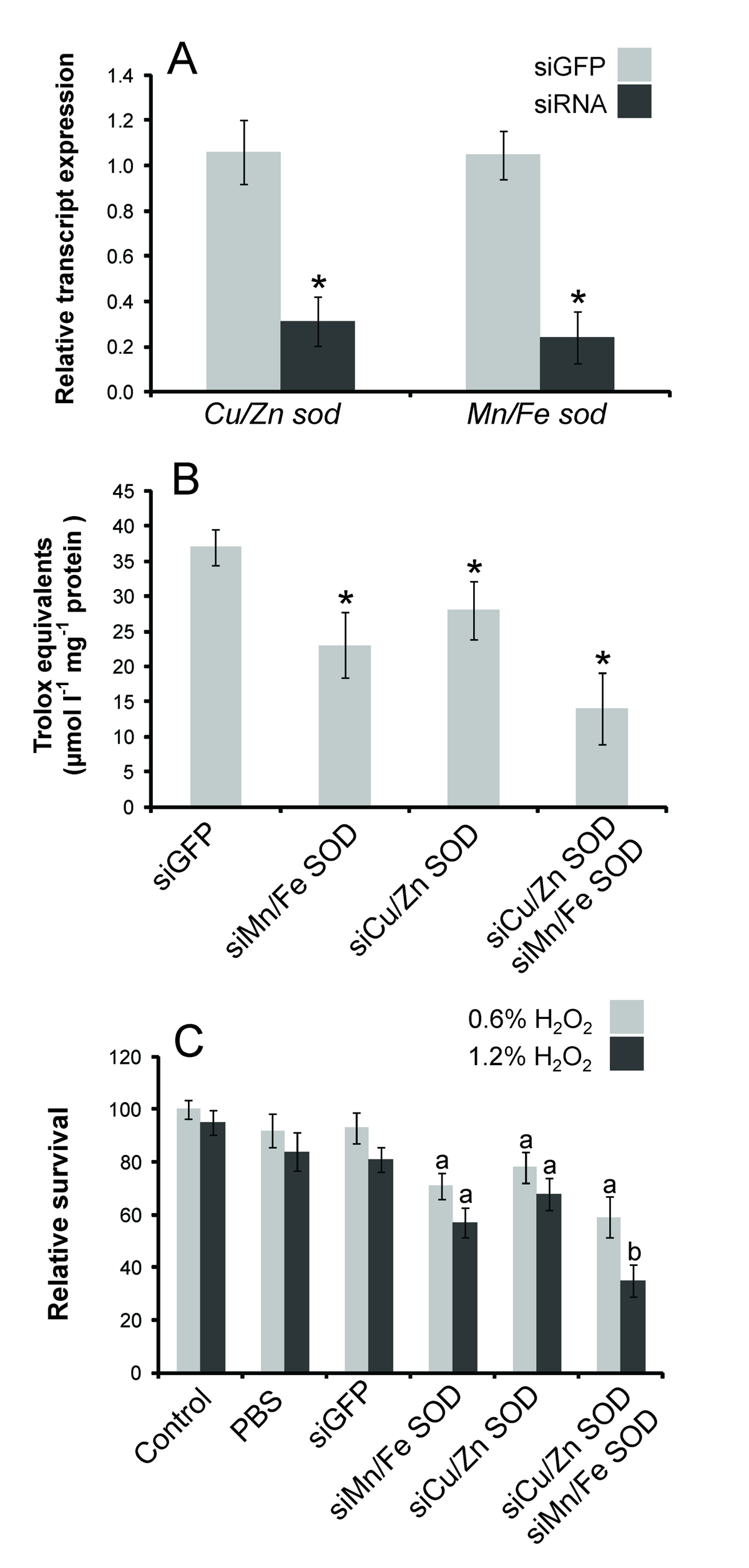

Supplement: Figure S4 — Reduction in milk gland protein levels after SOD gene knockdown. Tubulin was utilized as an internal control. Relative protein levels were determined with densitometry through the utilization of ImageJ. Mean ± SE of three blots. (TIF) [file pone.0087554.s004.tif]

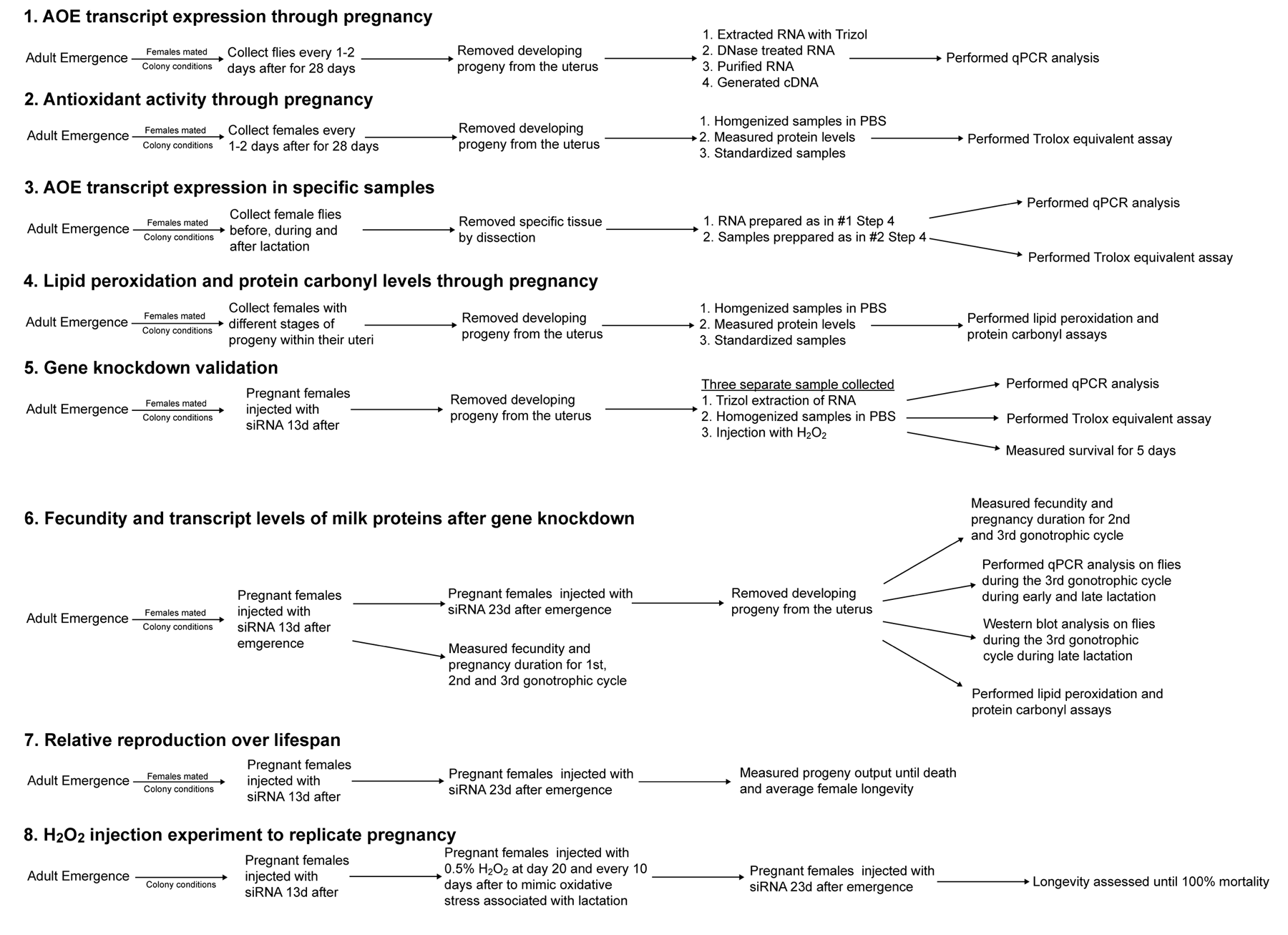

Supplement: Figure S5 — Diagram outlining experimental design. (TIF) [file pone.0087554.s005.tif]
